# Prevalence, morphology, and morphometry of the pterygospinous bar: a meta-analysis

**DOI:** 10.1007/s00276-019-02305-9

**Published:** 2019-08-28

**Authors:** Brandon Michael Henry, Przemysław A. Pękala, Paulina A. Frączek, Jakub R. Pękala, Konstantinos Natsis, Maria Piagkou, Krzysztof A. Tomaszewski, Iwona M. Tomaszewska

**Affiliations:** 1 Department of Anatomy, Jagiellonian University Medical College, International Evidence-Based Anatomy Working Group, 12 Kopernika St, 31–034 Kraków, Poland; 2 Department of Anatomy, Jagiellonian University Medical College, 12 Kopernika St, 31–034 Kraków, Poland; 3 Department of Anatomy and Surgical Anatomy, Faculty of Health Sciences, School of Medicine, Aristotle University of Thessaloniki, P.O. Box 300, 54124 Thessaloniki, Greece; 4 Department of Anatomy, School of Medicine, Faculty of Health Sciences, National and Kapodistrian University of Athens, 157 72 Athens, Greece; 5 Faculty of Medicine and Health Sciences, Andrzej Frycz Modrzewski Krakow University, Kraków, Poland; 6 Department of Medical Education, Jagiellonian University Medical College, 16 św. Łazarza St, 31-530 Kraków, Poland

**Keywords:** Pterygospinous bar, Pterygospinous foramen, Civinini’s foramen, Ossified ligament

## Abstract

**Purpose:**

The purpose of the study was to analyze the total prevalence, morphologic, and morphometric characteristics of the pterygospinous (PS) bar and its gender and ethnic differences among populations. PS bar is an ossified anatomic structure stretching between the posterior margin of the lateral pterygoid lamina to the angular spine of the undersurface of the sphenoid, with potential clinical implications. There is no consensus in the literature on its prevalence, morphologic, and morphometric characteristics.

**Methods:**

A thorough search of databases was conducted. Data on the prevalence, morphology, i.e., ossification type (complete and incomplete), side, gender, laterality, and morphometrics, of the PS bar were extracted and pooled into a meta-analysis.

**Results:**

A total of 35 studies (*n* = 14,047 subjects) were analyzed. The overall pooled prevalence of a complete PS bar was 4.4% (95% CI 3.7–5.1), while the overall pooled prevalence of an incomplete PS bar was significantly higher (11.6% [95% CI 8.5–15.2]). A complete PS bar was more prevalent among males and was more commonly unilaterally, on the left side.

**Conclusion:**

The overall prevalence of PS bar is quite common. It could be of importance for clinicians who should consider its potential presence when planning surgical approaches to the retropharyngeal and parapharyngeal space.

**Electronic supplementary material:**

The online version of this article (10.1007/s00276-019-02305-9) contains supplementary material, which is available to authorized users.

## Introduction

**Fig. 1:**
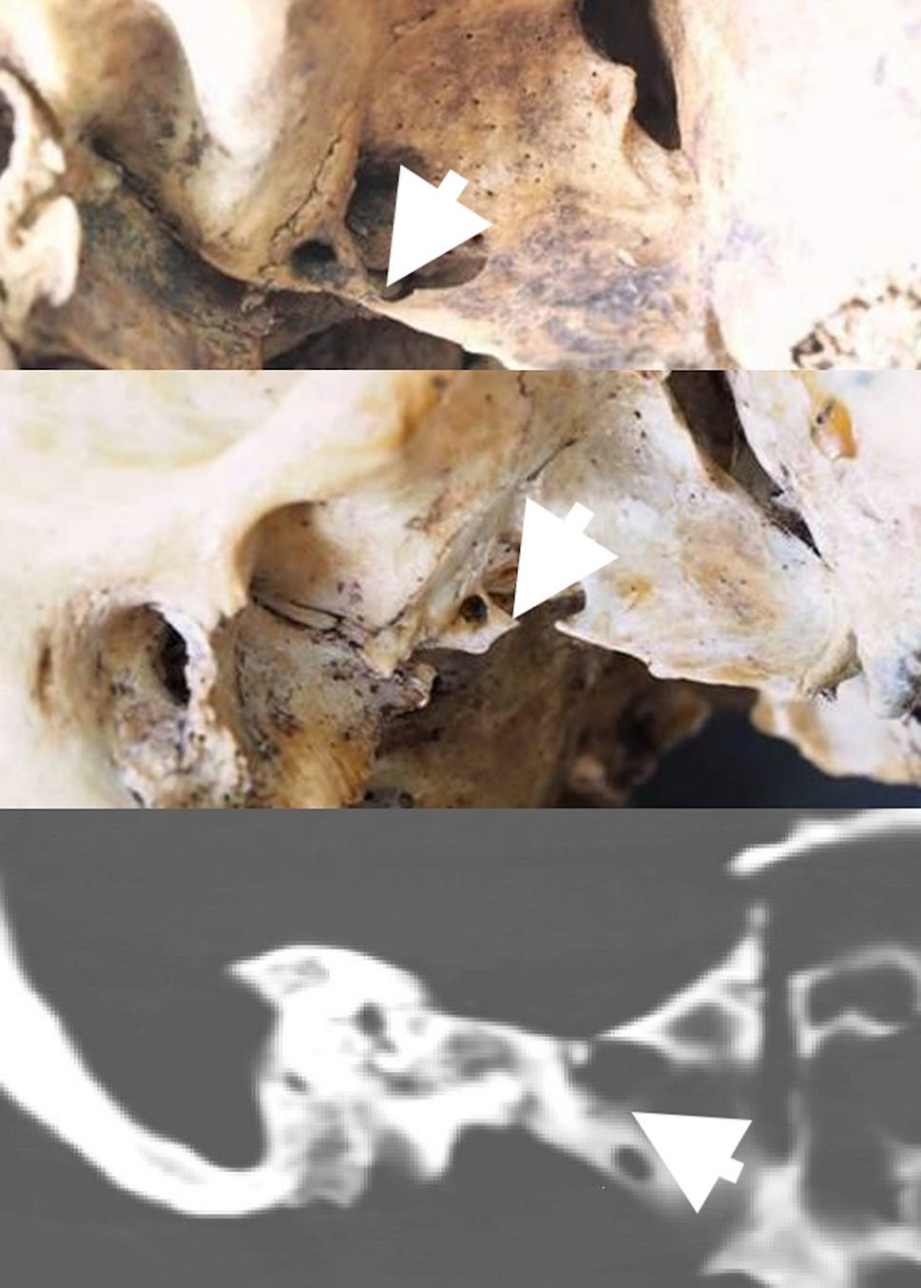
Lateral view of the dry skulls (**a**, **b**), and CT scan (**c**) with presentation of the **a**, **c** complete, and **b** incomplete pterygospinous bar (both marked with white arrows); *ANT* anterior, *POST* posterior

**Fig. 2:**
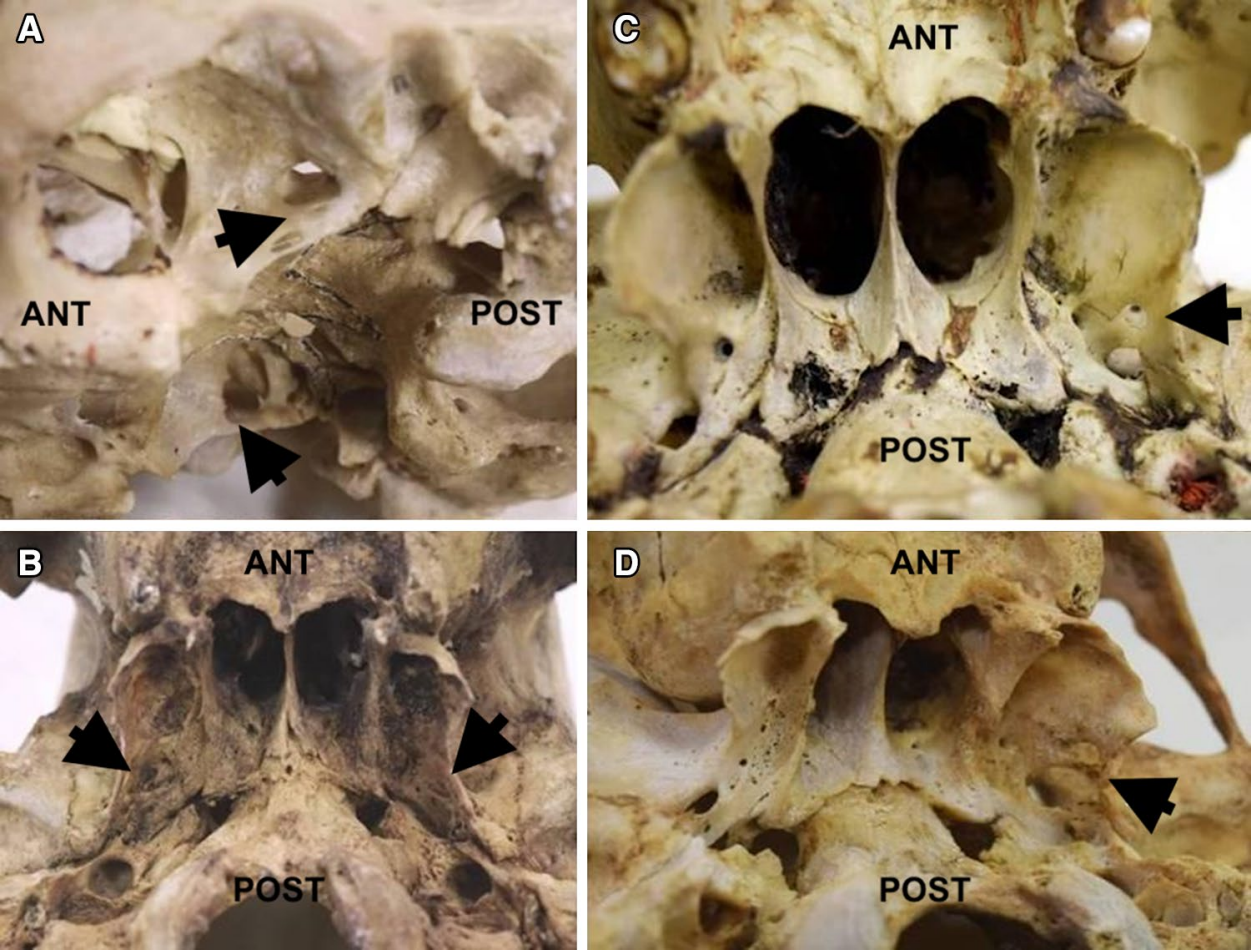
Lateral view (**a**) and inferior view (**b**–**d**) of the dry skulls with bilateral (**a**, **b**) and unilateral (**c**, **d**) complete pterygospinous bar left-sided presentation (all marked with black arrows); *ANT* anterior, *POST* posterior

**Fig. 3:**
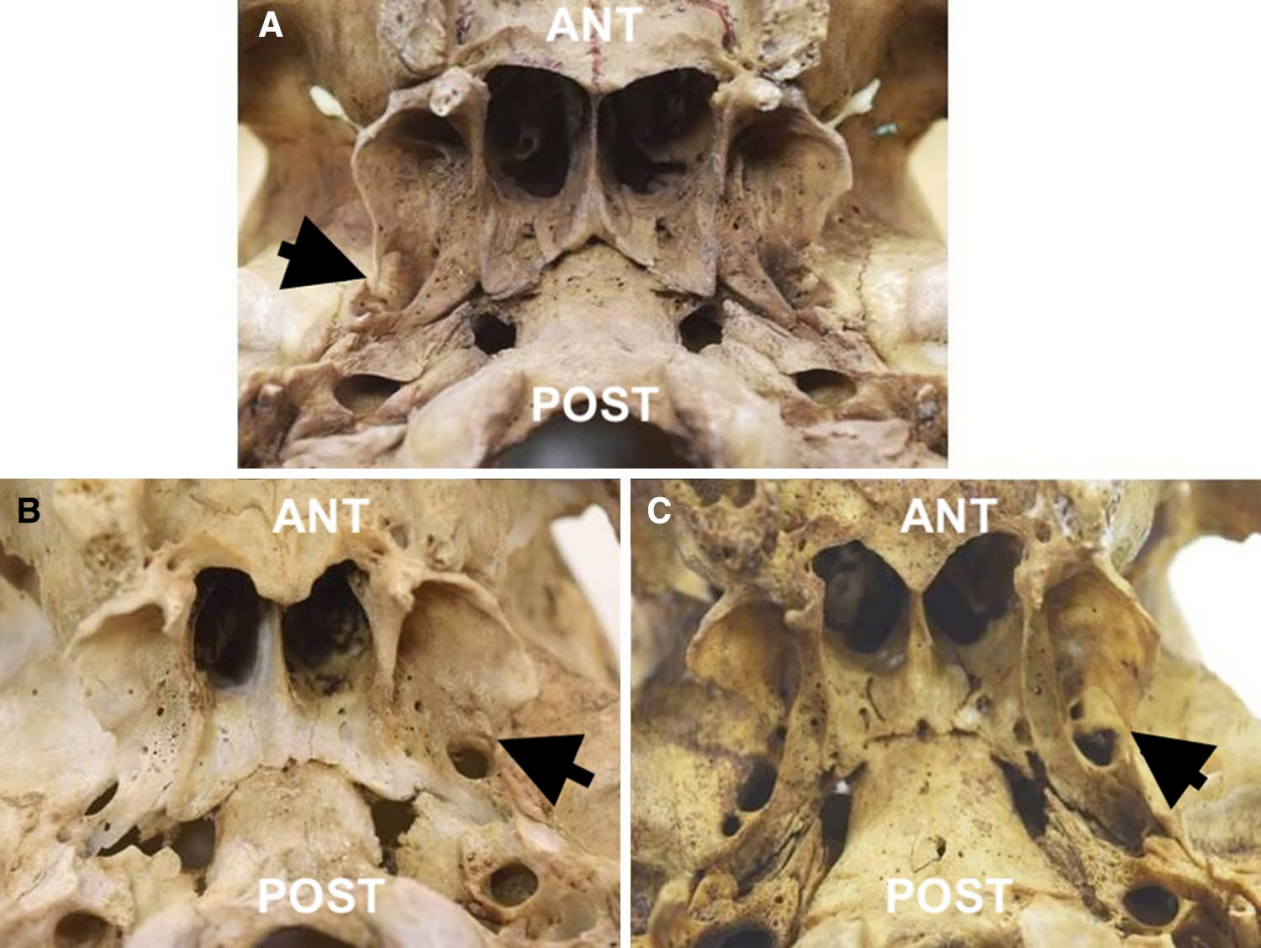
Inferior view of the base of the dry skulls with unilateral incomplete pterygospinous bar presenting on the left side (**b**, **c**) and right side (**a**) (all marked with black arrows); *ANT* anterior, *POST* posterior

The pterygospinous (PS) bar was first described by Fillipo Civinini in 1829, as an ossified structure stretching from the posterior free margin of the lateral pterygoid lamina to the angular spine of the undersurface of the greater wing of the sphenoid bone (Fig. 1) [5]. The PS bar can be present either uni- or bilaterally, and the extent of ossification can vary between sides. The following variants have been distinguished: bilateral complete, bilateral mixed (complete and incomplete), bilateral incomplete, unilateral complete, and unilateral incomplete [5, 25] (Figs. 2, 3). Both primary ossification and secondary ossification are considered as possible causes of PS bar formation [7]. The former theory is based on an observation of children’s skulls with still evident adjacent sutures, in which the PS bar was identified [2, 30]—suggesting a hereditary nature of this anatomical variant [19]. The secondary process refers to ossification of the PS ligament, which is the thickening of the fascia between the lateral and medial pterygoid muscles, that stretches between the spine of the sphenoid bone to the upper part of the posterior border of the lateral pterygoid plate, and which increases with aging [1, 2, 5]. This ligament may also be accompanied by an accessory PS ligament or, more rarely, it may be replaced by a muscular formation called the PS muscle, which inserts into the temporomandibular joint capsule [19, 25].

The prevalence of the PS bar is variable and inconsistent, ranging between 1% [8] and 31.2% [34]. Moreover, racial variations have been reported for a completely ossified PS bar, with a higher percentage of cases among Caucasians than Africans (10.7% vs 2.78%) [1]. A completely ossified PS bar may form a PS (or Civinini’s) foramen [19] through which passes the medial pterygoid vessels and nerve [30].

**Fig. 4:**
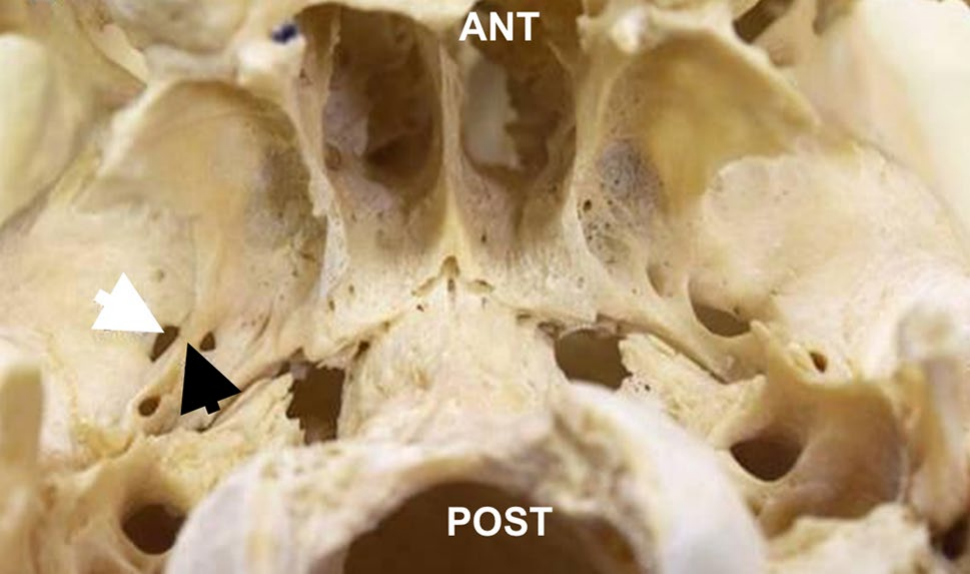
Inferior view of the base of the dry skull with a right-sided complete pterygoalar bar (marked with black arrow) medial to foramen ovale (marked with white arrow); *ANT* anterior, *POST* posterior

The PS bar is often confused with the pterygoalar (PA) bar [5, 26], as both bars are localized close to the foramen ovale (FO) area [9]. The PS bar may be found either below or medial to the FO, whereas the PA bar lies lateral to the FO or runs beneath it, dividing FO into two parts [19] (Fig. 4). The size of a PS foramen may vary even when occurring bilaterally. It may also occur as one large foramen (even up to 10 mm in diameter) or it may be divided into five distinctly separate foramina of variable size [5].

There is no consensus regarding which structures pass through a PS foramen. According to Goyal and Jain [13], the PS foramen transmits the mandibular nerve branches running to temporalis, masseter, and lateral pterygoid muscles. Peker et al. [30] reported that through the foramen pass the medial pterygoid vessels and nerve, whereas Chouke [5] mentioned the medial pterygoid nerve and some veins of the pterygoid venous plexus.

The structures formed by the ossification of the sphenoidal ligaments of the extracranial skull base may be clinically significant due to the risk of neurovascular compression and its possible resulting manifestations, such as trigeminal neuralgia [31, 40]. The presence of a PS bar is also of importance during surgeries in the retropharyngeal and parapharyngeal space, and in anaesthetic blockade, as the ossified structure may act as a barrier to the passage of the needle through the FO [5, 9, 13, 31].

The current study aimed to perform a comprehensive meta-analysis summarizing the total prevalence, morphologic and morphometric characteristics of the PS bar, and its probable racial and gender differences among the population.

## Methods

### Search strategy

To identify all studies that reported relevant information on the PS bar anatomy, an extensive search of the major electronic databases (PubMed, Embase, ScienceDirect, SciELO, BIOSIS, and Web of Science) was performed. The search was not restricted by any date or language. The following search terms were used: pterygospinous ligament OR Civinini bar OR pterygospinous bar OR foramen of Civinini OR pterygospinous foramen. An additional search through the references of all identified studies was conducted to identify other potentially eligible articles. The Preferred Reporting Items for Systematic Reviews and Meta-Analyses (PRISMA) guidelines were strictly followed during this study (Supplement 1).

### Eligibility assessment

Three independent reviewers (PAF, JRP, and PAP) assessed the eligibility of each study for inclusion into the meta-analysis. The inclusion criteria were peer-reviewed, cadaveric or imaging studies reporting extractable data on the prevalence, morphologic, and morphometric characteristics of the PS bar. Any studies published in languages other than English were translated by medical professionals fluent both in English and the original language of the study and their eligibility for the inclusion was further assessed by the authors. Case studies, reviews, letters to editors, conference abstracts, or studies containing incomplete or irrelevant data were excluded. Any issues during the eligibility assessment were resolved by a unanimous consensus of all the authors.

### Data extraction

Three independent reviewers (PAF, JRP, and PAP) extracted the relevant data. The extracted data included year of study, geographical location, type of the study (cadaveric and radiological), prevalence and ossification type (complete and incomplete) of the PS bar, side of occurrence, gender dimorphism, laterality, and morphometric details of the PS bar.

A complete PS bar was defined as the bony bridge between the lateral pterygoid plate and the sphenoidal spine. Any deviation from the above referred variant was identified as an incomplete PS bar. The mean horizontal and vertical diameters of the PS foramen and the mean length and width of the PS bar were extracted from cadaveric studies, whenever possible. In case of any problem with data in the articles, the authors of the included studies were contacted for clarification.

### Statistical analysis

MetaXL 2.0 by EpiGear International Pty Ltd (Wilston, Queensland, Australia) was used to calculate pooled prevalence estimates of the PS bar. The morphometric data analysis was performed using Comprehensive Meta-Analysis version 3.0 by Biostat (Englewood, New Jersey, USA) to calculate pooled means. All analyses used a random effects model. The heterogeneity of the included studies was assessed with the Chi-square test and *I*^2^ statistic. Cochran’s *Q p* value < 0.10 in Chi-square test indicated significant heterogeneity among studies. The following intervals were used to interpret the *I*^2^ statistic: 0–40%—“might not be important”, 30–60%—“might indicate moderate heterogeneity”, 50–90%—“may indicate substantial heterogeneity”, 75–100%—“may represent considerable heterogeneity” [17]. Subgroup analysis by the type of study, gender, side (left vs. right), laterality, and geographical region (continent, country) was performed to identify the sources of heterogeneity. To additionally probe the source of heterogeneity, a sensitivity analysis of studies with sample size equal to or greater than 500 subjects, when appropriate. Confidence intervals were compared between the groups to identify statistically significant differences. Overlap between the confidence intervals suggested that the differences between groups were statistically insignificant [16].

## Results

### Study identification

**Fig. 5:**
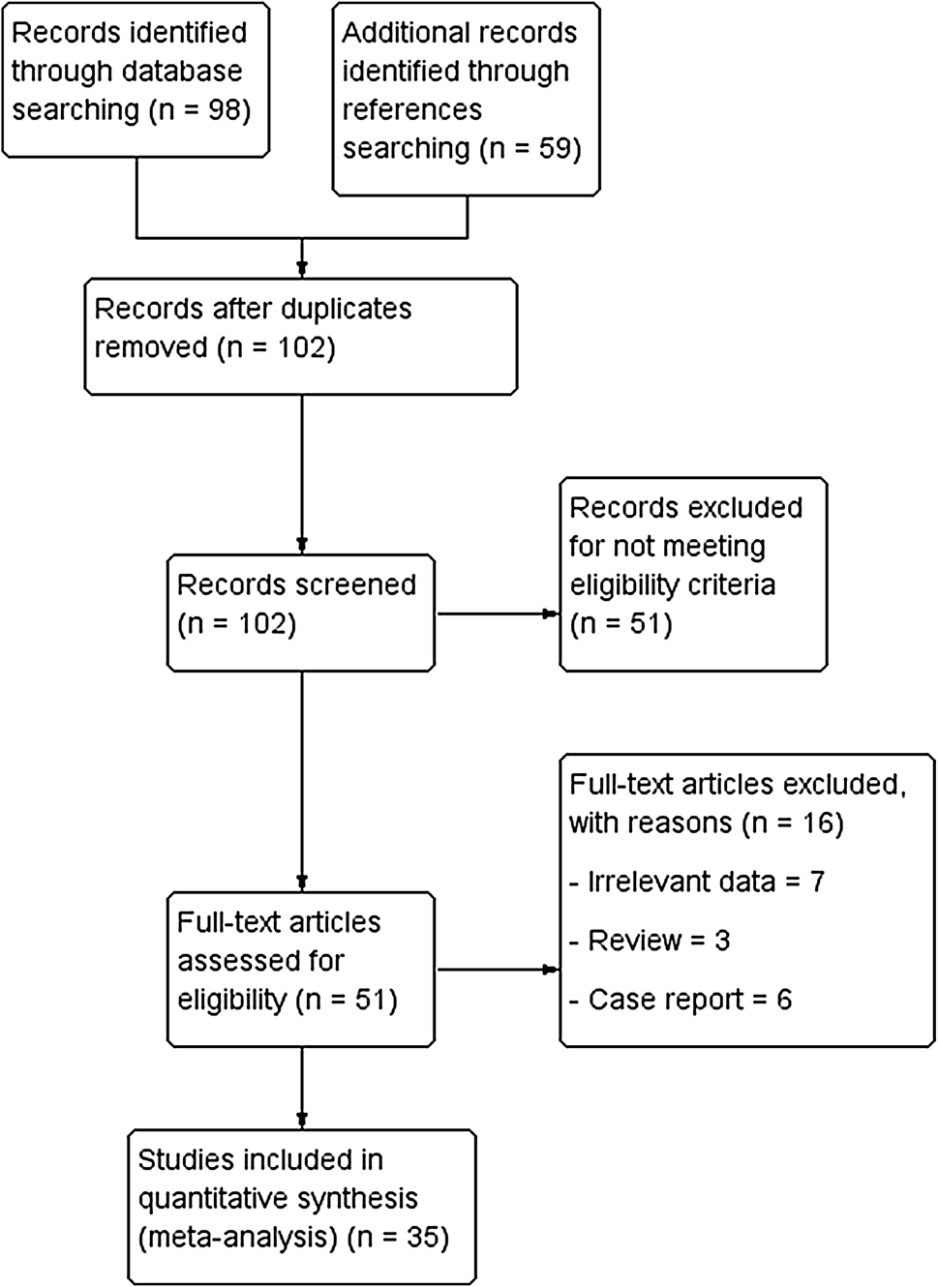
Flowchart of studies through the meta-analysis

The study identification procedure is presented in Fig. 5.

An extensive search of the major electronic databases yielded a total of 98 articles. The search through the references of included studies provided additional 59 articles. Records that were duplicates and not meeting eligibility criteria were excluded. Thus, a total of 51 full text articles were assessed by authors for potential eligibility. Finally, 35 articles were deemed eligible and included into the meta-analysis.

### Characteristics of included studies

The characteristics of included studies are presented in Table 1. A total of 35 studies (*n* = 14,047 subjects), 34 cadaveric (*n* = 13,954), and 1 radiological (radiographs of dry skulls) (*n* = 93 subjects) were included into the meta-analysis. Among the included articles, the oldest study was conducted in 1875 [25] and the most recent in 2016 [13]. The included studies originated from variable geographical regions, such as Asia, Europe, North America, and South America, with 14 studies (*n* = 2776 subjects) conducted in India.

**Table 1:** Characteristics of included studies with their prevalence of the pterygospinous (PS) bar (complete and incomplete)

Author(s)	Year	Population	Study	Subjects	% Prevalence of complete PS bar (number of complete PS bar)	% Prevalence of incomplete PS bar (number of incomplete PS bar)
Macalister [25]	1875	Irish	C	144	9.7 (14)	0 (0)
Roth [35]	1882	Germans	C	326	7.4 (24)	16.0 (52)
von Brunn [3]	1891	Germans	C	406	5.2 (21)	18.5 75)
Grosse [14]	1893	Germans	C	400 600	3.0 (12) 2.3 (14)	20.0 (80) 10.0 (60)
LeDouble [23]	1903	French	C	1535	4.4 (67)	No data
Oetteking [29]	1930	Americans	C	467	6.2 (29)	33.0 (151)
Chouke [5, 6]	1946 1947	Americans	C	1544	6.25 (97)	0 (0)
				2745	4.7 (128)	23.1 (633)
Priman and Etter [33]	1959	Americans	C	250	3.2 (8)	8.0 (20)
Tebo [43]	1968	Indians	C	516	3.9 (20)	32.9 (170)
Dodo [10]	1974	Japanese	C	329	5.5 (18)	0 (0)
Dodo and Ishida [11]	1987	Japanese		1160	6.6 (77)	No data
Shaw [40]	1993	Indians	C	454	4.4 (20)	11.7 (53)
Krmpotic-Nemanic et al. [20]	1999	Croatians	C	120	4.2 (5)	0 (0)
Kapur et al. [18]	2000	Bosnians and Herzegovinians	C	305	3.6 (11)	14.8 (45)
Saiki [37]	2000	Japanese	C	91	4.4 (4)	No data
Ludinghausen et al. [46]	2006	Japanese	C	100	6.0 (6)	11.0 (11)
Das and Paul [8]	2007	Indians	C	50	0 (0)	2.0 (1)
Nayak et al. [28]	2007	Indians (Dravidian)	C	416	5.8 (24)	3.8 (16)
Antonopolou et al. [2]	2008	Greeks	C	50	16.0 (8)	22.0 (11)
Tubbs et al. [44]	2009	Americans	C	152	0.7 (1)	0.7 (1)
Suazo et al. [42]	2010	Brazilians	C	312	1.6 (5)	13.1 (41)
Rosa et al. [34]	2010	Brazilians	XR	93	8.6 (8)	19.4 (18)
Sharma and Garud [39]	2011	Indians	C	50	2.0 (1)	0 (0)
Shinde et al. [41]	2011	Indians (Karnataka)	C	65	0 (0)	3.1 (2)
Devi Jansirani et al. [9]	2012	Indians	C	204	1.0 (2)	10.8 (22)
Chakravarthi et al. [4]	2013	Indians (Karnataka)	C	100	3.0 (3)	1.0 (1)
Saran et al. [38]	2013	Indians (Chennai)	C	80	2.5 (2)	11.3 (9)
Verma et al. [45]	2013	Indians (UP)	C	116	12.9 (15)	1.7 (2)
Kavitha et al. [19]	2014	Indians	C	100	1.0 (1)	16.0 (16)
Yadav et al. [48]	2014	Indians (UP)	C	50 500	4.0 (2) 4.0 (20)	10.0 (5) 6.2 (31)
Goyal and Jain [13]	2016	Indians (Punjab)	C	75	2.7 (2)	14.7 (11)
Ryu et al. [36]	2016	South Koreans	C	142	2.1 (3)	24.6 (35)

*C* Cadaveric, *XR* radiograph

### Prevalence of the complete PS bar

**Fig. 6:**
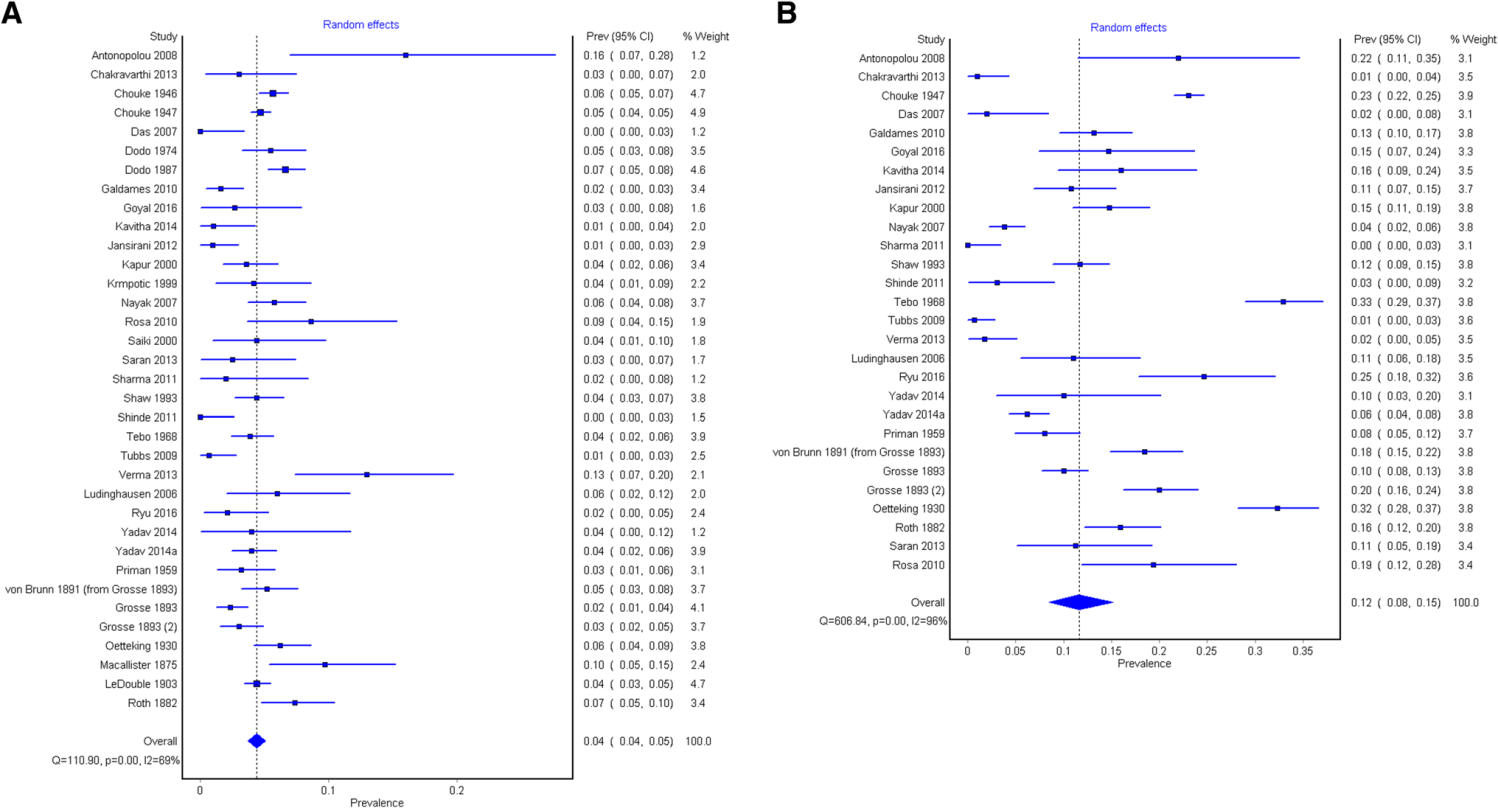
Forest plots for the population pooled prevalence of the complete and incomplete pterygospinous (PS) bar

A total of 35 studies (*n* = 14,047 subjects) reported data on the prevalence of a complete PS bar (Fig. 6). The overall pooled prevalence of the complete bar was 4.4% (95% CI 3.7–5.1). In subgroup analysis, the pooled prevalence in cadaveric studies was 4.3% (95% CI 3.6–5.0).

Geographical analysis showed that a complete PS bar is most often found in Europe with a pooled prevalence of 4.9% (95% CI 3.7–6.4), followed by South (4.5% [95% CI 0.0–12.9]) and North America (4.4% [95% CI 3.2–5.8]) (Table 2). However, the differences were not significant. The pooled prevalence of the complete PS bar was the lowest in Asia (3.7% [95% CI 2.6–5.0]). Among Asian countries, the PS bar was significantly more prevalent in Japan (6.3% [95% CI 5.2–7.5]), than in India (3.0% [95% CI 1.7–4.6]).

**Table 2:** Geographical subgroups, gender, and sensitivity analysis for complete pterygospinous (PS) bars. Sensitivity analyses were conducted on studies with more than 500 patients

Subgroup	Number of studies (number of subjects)	Pooled prevalence of complete PS bar: % (95% CI)	*I*^2^: % (95% CI)	Cochran’s *Q*, *p* value
Overall studies	35 (14,047)	4.4 (3.7–5.1)	69.34 (56.69–78.30)	*p* < 0.001
Cadaveric studies	34 (13,954)	4.3 (3.6–5.0)	69.44 (56.62–78.47)	*p* < 0.001
Sensitivity	7 (8600)	4.6 (3.7–5.5)	72.93 (41.74–87.42)	0.001
North Americans	5 (5158)	4.4 (3.2–5.8)	73.16 (32.89–89.26)	0.005
Asians	18 (4144)	3.7 (2.6–5.0)	71.33 (53.85–82.19)	*p* < 0.001
Europeans	10 (4340)	4.9 (3.7–6.4)	71.65 (46.11–85.09)	*p* < 0.001
South Americans	2 (405)	4.5 (0.0–12.9)	88.33 (55.37–96.95)	0.003
Brazilians	2 (405)	4.5 (0.0–12.9)	88.33 (55.37–96.95)	0.003
Germans	4 (1732)	4.2 (2.3–6.6)	79.88 (46.68–92.41)	0.002
Indians	13 (2322)	3.0 (1.7–4.6)	71.11 (49.24–83.56)	*p* < 0.001
Japanese	4 (1680)	6.3 (5.2–7.5)	0.0 (0.0–49.20)	0.8240
Americans	5 (5158)	4.4 (3.2–5.8)	73.16 (32.89–89.26)	0.005
Males	4 (3771)	5.7 (4.9–6.4)	0.38 (0.00–84.75)	0.390
Females	4 (1076)	2.4 (1.5–3.6)	14.47 (0.00–86.90)	0.320

The subgroup analysis with respect to gender showed that the complete PS bar was found to be significantly more prevalent among males, with a pooled prevalence of 5.7% (95% CI 4.9–6.4), than females (2.4% [95% CI 1.5–3.6]) (Table 2).

A total of 23 studies (*n* = 1438 subjects) were included in the analysis of the laterality of the PS bar (Table 4) and 20 studies (*n* = 407 subjects) reported data on the complete PS bar in relation to side of occurrence (Table 5). The most common configuration of the PS bar was unilateral, with a pooled prevalence of 23.7% (95% CI 10.0–36.2), followed by bilateral (8.0% [95% CI 0.8–18.0]), although the differences were not significant. When a complete PS bar was observed, it was found on the left side in 53.1% (95% CI 46.0–60.1) of cases and on the right side in 46.9% (95% C: 39.9–54.0) of cases.

To further probe the source of heterogeneity, a sensitivity analysis was conducted on studies with a sample size of more than 500 subjects, which included 7 studies (*n* = 8600 subjects). The pooled prevalence in this group was 4.6% (95% CI 3.7–5.5).

### Prevalence of the incomplete PS bar

A total of 28 studies (*n* = 9124 subjects) were included in the analysis on the prevalence of an incomplete PS bar (Fig. 6). The overall pooled prevalence of an incomplete PS bar was significantly higher than a complete PS bar and amounted to 11.6% (95% CI 8.5–15.2) (Table 3). The subgroup analysis by study type showed a pooled prevalence of 11.4% (95% CI 8.2–15.0) (Table 3) in cadaveric studies.

**Table 3:** Geographical subgroups, gender, and sensitivity analysis for incomplete ossified pterygospinous (PS) bars

Subgroup	Number of studies (number of subjects)	Pooled prevalence of incomplete PS bar % (95% CI)	*I*^2^: % (95% CI)	Cochran’s *Q*, *p* value
Overall studies	28 (9124)	11.6 (8.5–15.2)	95.55 (94.45–96.43)	*p* < 0.001
Cadaveric studies	27 (9031)	11.4 (8.2–15.0)	95.71 (94.64–96.57)	*p* < 0.001
Sensitivity	4 (4361)	16.2 (7.0–28.0)	98.47 (97.57–99.04)	*p* < 0.001
North Americans	4 (3614)	12.6 (3.3–25.8)	98.12 (96.90–98.86)	*p* < 0.001
Asians	15 (2564)	8.4 (3.9–14.3)	95.09 (93.27–96.42)	*p* < 0.001
Europeans	7 (2541)	15.4 (12.2–18.8)	80.27 (59.89–90.30)	*p* < 0.001
South Americans	2 (405)	15.3 (9.8–21.8)	54.02 (0.00–88.69)	0.140
Brazilians	2 (405)	15.3 (9.8–21.8)	54.02 (0.00–88.69)	0.140
Germans	4 (1732)	15.7 (11.1–21.0)	87.75 (70.94–94.84)	*p* < 0.001
Indians	13 (2322)	7.0 (2.6–13.2)	95.48 (93.69–96.76)	*p* < 0.001
Americans	4 (3614)	12.6 (3.3–25.8)	98.12 (96.90–98.86)	*p* < 0.001
Males	2 (2385)	20.7 (14.4–27.9)	78.22 (5.16–95.00)	0.032
Females	2 (589)	16.2 (9.7–23.9)	75.67 (0.00–94.48)	0.043

The subgroup analysis with respect to geographical location showed variable prevalence of the incomplete PS bar. The analysis revealed that the incomplete PS bar was most common among Europeans (15.4% [95% CI 12.2–18.8]), followed by South Americans (15.3% [95% CI 9.8–21.8]) and North Americans (12.6% [95% CI 3.3–25.8]), with the lowest pooled prevalence found in Asians (8.4% [95% CI 3.9–14.3]), though the differences were not significant (Table 3).

Subgroup analysis in relation to laterality and with respect to side included 23 studies (*n* = 1438 subjects) (Table 4) and 18 studies (*n* = 1238 subjects) (Table 5), respectively. The incomplete PS bar was more often observed in unilateral configuration (45.3% [95% CI 26.4–57.4]) and on the left side (50.8% [95% CI 47.4–54.2]), followed by bilateral appearance (19.3% [95% CI 7.0–31.4]) and on the right side (49.2% [95% CI 45.8–52.6]). An additional sensitivity analysis was conducted including studies with a sample size greater than 500. The pooled prevalence of the incomplete PS bar in this group was 16.2% (95% CI 7.0–28.0) (Table 3).

**Table 4:** Analysis of laterality of the pterygospinous (PS) bar

Number of studies (subjects with PS bar)	Unilateral complete % (95% CI)	Unilateral incomplete % (95% CI)	Bilateral complete % (95% CI)	Bilateral incomplete % (95% CI)	Mixed^a^: % (95% CI)	*I*^2^: % (95% CI)^b^
23 (1438)	23.7 (10.0–36.2)	45.3 (26.4–57.4)	8.0 (0.8–18.0)	19.3 (7.0–31.4)	3.7 (0.0–10.2)	95.4 (94.1–96.4)

Subgroup	Number of studies (subjects with PS bar)	Right-sided PS: % (95% CI)	Left-sided PS: % (95% CI)	*I*^2^: % (95% CI)	Cochran’s *Q*, *p* value
Complete PS	20 (407)	46.9 (39.9–54.0)	53.1 (46.0–60.1)	24.95 (0.00–56.47)	0.150
Incomplete PS	18 (1238)	49.2 (45.8–52.6)	50.8 (47.4–54.2)	4.96 (0.00–52.45)	0.396

^a^Mixed type—both a complete PS on one side and an incomplete PS on the other

^b^Cochran’s *Q*, *p* value for all groups < 0.001

**Table 5:** Prevalence of complete and incomplete pterygospinous (PS) ligaments with respect to side of occurrence

Subgroup	Number of studies (subjects with PS bar)	Right-sided PS: % (95% CI)	Left-sided PS: % (95% CI)	*I*^2^: % (95% CI)	Cochran’s *Q*, *p* value
Complete PS	20 (407)	46.9 (39.9–54.0)	53.1 (46.0–60.1)	24.95 (0.00–56.47)	0.150
Incomplete PS	18 (1238)	49.2 (45.8–52.6)	50.8 (47.4–54.2)	4.96 (0.00–52.45)	0.396

### Morphometric analysis of the complete PS bar and foramen

Two cadaveric studies (*n* = 89 subjects) were included in the analysis on the horizontal diameter and four cadaveric studies (*n* = 137 subjects) on the vertical diameter of the complete PS foramen. The pooled mean horizontal and vertical diameters of the PS foramen were 9.05 mm (95% CI 5.99–12.11) and 5.75 mm (95% CI 3.97–7.53), respectively (Table 6). Three cadaveric studies (*n* = 75 subjects) reported extractable data on the length and width of the PS bar. The pooled mean dimensions of the PS bar were as follows: 7.48 mm (95% CI 4.69–10.28) in length and 3.06 mm (95% CI 2.38–3.74) in width.

**Table 6:** Morphometric analysis of the pterygospinous (PS) bar

Diameters of PS structures	Number of cadaveric studies (number of PS)	Pooled mean distance: mm (95% CI)	*I*^2^: %
Foramen			
Horizontal	2 (89)	9.05 (5.99–12.11)	0.0
Vertical	4 (137)	5.75 (3.97–7.53)	0.0
Bar			
Length	3 (75)	7.48 (4.69–10.28)	0.0
Width	3 (75)	3.06 (2.38–3.74)	0.0

## Discussion

This study aimed to perform a comprehensive meta-analysis of the prevalence and morphometry of the complete and incomplete PS bars, based on more than 14,000 subjects. In this study, we found that the PS bar is relatively common. The pooled prevalence of the complete type was 4.4%, while that of the incomplete type was 11.6%. The general tendency that the incomplete PS bar is significantly more prevalent than the complete one has been systematically reported in previous works [2, 7, 9, 13, 18, 19, 32].

Some of the former studies [7, 9] found left-side predominance, while others [32, 48] found the PS bar to be more common on the right side. In our analysis, the pooled prevalence of unilateral configuration for complete and incomplete PS bars was 23.7% and 45.3%, respectively, whereas in 31% of the cases, the PS bar was identified bilaterally. Thus, we would suggest that anesthesiologists and surgeons take particular caution during surgeries requiring bilateral access to the retropharyngeal and parapharyngeal regions, as the PS bar presence may be expected on both sides.

Analysis with respect to gender dimorphism revealed that the complete PS bar is significantly more prevalent in males than females. At the same time, no significant differences between genders were found for the incomplete PS bar. The PS bar predominance in males has been reported previously [7, 10], but the literature lacks information on the possible reasons for this gender discrepancy.

The presence of ossified PS ligament has been suggested to play a role in several entrapment syndromes [12, 27, 31, 32]. The lingual nerve travels between the medial pterygoid muscle and PS ligament, and therefore, in the presence of ossified PS ligament, it can be compressed against PS bar, which can lead to clinical symptoms. Moreover, the PS bar can separate fibers of lingual nerve and divide it into anterior and posterior parts [12]. In such cases, the posterior part of lingual nerve traverses lateral to the PS bar, while the anterior part passes medially between the tensor veli palatini and PS bar, thus being more prone to entrapment [12]. The clinical manifestation includes mandibular pain, numbness, and/or altered sensation in the anterior two-thirds of the tongue. The compression of other branches of mandibular nerve has also been reported [31, 44]. Moreover, as the chorda tympani of the facial nerve runs with the lingual nerve, it can also be compressed against PS bar and result in impaired taste and salivary function [32]. Shaw [40] discussed that the bone overgrowth below the FO might be a potential mechanism of trigeminal neuralgia in patients with a PS bar. He also hypothesized that the accessory meningeal artery may be angulated when it passes around bony variants below the FO. This could lead to disturbances in blood flow and neuronal ischemic damage in the trigeminal ganglion [40]. Therefore, radiologic identification of PS bar might aid in determining the potential etiology of trigeminal neuralgia in patients in which the cause of the symptoms is not clearly evident. Radiologically, the PS bar can appear as duplicated or bifurcated foramen ovale [31]. While CT scan of the cranial base ensures proper visualization of anatomical relations in the region, radiographic scan is often sufficient. The Hirtz axial radiograph and submentovertex projection allow identification of the PS bar as well as other structures at the skull base [34].

The PS bar can reduce the space between the lateral pterygoid plate and the sphenoidal spine [9]. Thus, this narrow space can limit surgical access to the retropharyngeal and parapharyngeal space. It was suggested that the ossified variant may preclude the trigeminal ganglion thermo-coagulation [8]. Moreover, in cases when the PS bar is located inferiorly to the FO, caution should be taken during anaesthetic procedures of the trigeminal ganglion, since the ossified ligament may act as an obstacle for the needle. If the surgeon has difficulty accessing the foramen ovale with the needle despite using different angles, the presence of PS bar should be considered. In such cases, the surgeon may abort the procedure and conduct CT scan or radiographic scan of the skull base postoperatively to identify the obstacle. If the PS bar is present, intraoperative CT-guided neuronavigation could be utilized to guide the needle around the bony bridge [44]. Otherwise, an inframandibular approach to the trigeminal ganglion should be considered.

Although some authors [15, 24, 30] have suggested that the PS bar may be a result of secondary ossification of the PS ligament, the fact that the PS bar is more common in skulls of other mammals (Old World monkeys, foxes, roes, and rabbits) indicates that it may be a phylogenetic remnant [46]. Moreover, the variable geographical prevalence of the PS bar detected in the current study and the racial differences reported earlier [7, 21] indicate its genetic and primary origin. In addition, suggestive of this type of etiology was the study by Lang and Hetterlich [22] who described the PS bar presence in skulls of 5 year old children with still evident adjacent sutures.

The main limitation of this meta-analysis was the considerable heterogeneity among the included studies. Not all studies reported data on gender dimorphism, laterality or the side of PS bar occurrence, which limited assessment of these secondary outcomes and led to distortion of some of the results, such as finding the overall prevalence of PS bar lower than in either of the analyzed gender subgroups. In addition, the literature lacked a strictly determined definition of the term “incomplete PS”, with no information on the extent of ossification of the PS ligament required for being called a PS bar. Moreover, there was a shortage of radiological studies (only one study was performed on dry skulls) and only a few studies reported morphometric data of the PS bar. Thus, future radiological studies and studies that will additionally focus on the etiology and morphometry of the PS bar are needed to fully understand the role of the ossified variant. Finally, the geographical analysis was limited by the lack of prevalence data from Africa.

However, despite all limitations listed above, this is the most comprehensive study on the PS bar. Meta-analysis design allowed to pool result of studies conducted since 1875 and perform combined analysis. In addition, to minimize bias of included studies, we used the AQUA Tool which was specifically designed for anatomical meta-analyses. Throughout the process of conducting this study, authors strictly followed PRISMA guidelines. All the undertaken actions contributed to minimization of the bias of this meta-analysis.

## Conclusion

In conclusion, in the current study, we found that the PS bar is relatively common and a predominance of the complete PS bar in males was observed. Thus, acknowledging the potential clinical significance of this ossified structure, clinicians should consider its potential presence during planning approaches to retropharyngeal and parapharyngeal space, as well as when performing trigeminal ganglion block.

## Electronic supplementary material

Below is the link to the electronic supplementary material.

The Anatomical Quality Assessment (AQUA) Tool (DOCX 39 kb)
