# Supplementary material for: Prevalence, morphology, and morphometry of the pterygospinous bar: a meta-analysis
Source: Surg Radiol Anat. 2019 Aug 28;42(5):497–507. doi: 10.1007/s00276-019-02305-9 (PMC7096365; doi:10.1007/s00276-019-02305-9)
Supplement: Supplementary file 1 — The Anatomical Quality Assessment (AQUA) Tool (DOCX 39 kb) [file 276_2019_2305_MOESM1_ESM.docx]

**Supplement 1**

**Prevalence, Morphology and Morphometry of the Pterygospinous Bar: a Meta-Analysis**

Brandon Michael Henry, Przemysław A. Pękala, Paulina A. Frączek*, Jakub R. Pękala, Konstantinos Natsis, Maria Piagkou, Krzysztof A. Tomaszewski, Iwona M. Tomaszewska

^*International Evidence – Based Anatomy Working Group, Department of Anatomy, Kopernika 12, Kraków, Poland;^ [^paulinafraczek2@gmail.com^](mailto:paulinafraczek2@gmail.com)

***AQUA Tool – Pterygospinous Bar: A meta-analysis***

| **STUDY** | **RISK OF BIAS** | | | | |
| --- | --- | --- | --- | --- | --- |
|  | **OBJECTIVE(S) AND STUDY CHARACTERISTICS** | **STUDY DESIGN** | **METHODOLOGY CHARACTERIZATION** | **DESCRIPTIVE ANATOMY** | **REPORTING OF RESULTS** |
| Macalister (1875) | 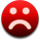 | 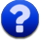 | 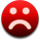 | 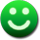 | 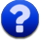 |
| Roth (1882) | 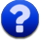 | 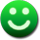 | 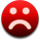 | 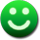 | 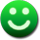 |
| von Brunn (1891) | 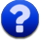 | 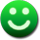 | 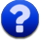 | 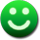 | 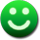 |
| Grosse (1893) | 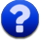 | 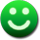 | 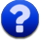 | 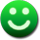 | 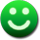 |
| LeDouble (1903) | 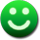 | 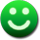 | 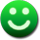 | 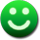 | 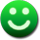 |
| Oetteking (1930) | 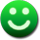 | 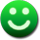 | 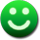 | 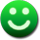 | 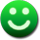 |
| Chouke (1946) | 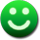 | 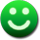 | 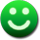 | 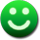 | 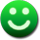 |
| Chouke (1947) | 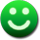 | 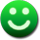 | 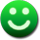 | 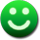 | 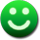 |
| Priman and Etter (1959) | 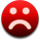 | 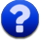 | 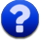 | 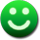 | 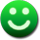 |
| Tebo (1968) | 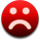 | 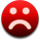 | 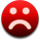 | 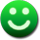 | 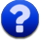 |
| Dodo (1974) | 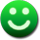 | 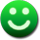 | 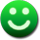 | 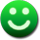 | 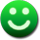 |
| Dodo and Ishida (1987) | 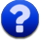 | 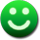 | 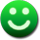 | 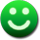 | 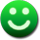 |
| Shaw (1993) | 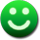 | 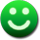 | 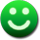 | 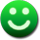 | 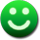 |
| Krmpotic-Nemanic et al. (1999) | 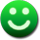 | 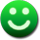 | 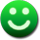 | 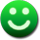 | 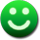 |
| Kapur et al. (2000) | 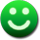 | 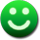 | 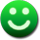 | 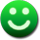 | 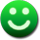 |
| Saiki (2000) | 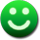 | 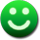 | 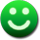 | 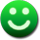 | 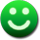 |
| Ludinghausen et al. (2006) | 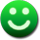 | 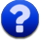 | 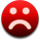 | 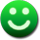 | 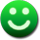 |
| Das and Paul (2007) | 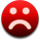 | 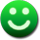 | 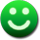 | 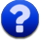 | 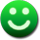 |
| Nayak et al. (2007) | 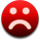 | 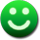 | 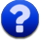 | 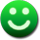 | 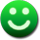 |
| Antonopolou et al. (2008) | 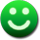 | 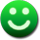 | 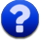 | 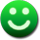 | 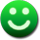 |
| Tubbs et al. (2009) |  |  |  |  |  |
| Suazo et al. (2010) |  |  |  |  |  |
| Rosa et al. (2010) |  |  |  |  |  |
| Sharma and Garud (2011) |  |  |  |  |  |
| Shinde et al. (2011) |  |  |  |  |  |
| Jansirani et al. (2012) |  |  |  |  |  |
| Chakravarthi et al. (2013) |  |  |  |  |  |
| Saran et al. (2013) |  |  |  |  |  |
| Verma et al. (2013) |  |  |  |  |  |
| Kavitha et al. (2014) |  |  |  |  |  |
| Yadav et al. (2014) |  |  |  |  |  |
| Goyal and Jain (2016) |  |  |  |  |  |
| Ryu et al. (2016) |  |  |  |  |  |
